# Prudence, pleasure, and cognitive ageing: Configurations of the uses and users of brain training games within UK media, 2005–2015

**DOI:** 10.1016/j.socscimed.2017.06.028

**Published:** 2017-08

**Authors:** Martyn Pickersgill, Tineke Broer, Sarah Cunningham-Burley, Ian Deary

**Affiliations:** a Usher Institute of Population Health Sciences and Informatics, University of Edinburgh, United Kingdom; b Centre for Cognitive Ageing and Cognitive Epidemiology, University of Edinburgh, United Kingdom

**Keywords:** United Kingdom, Brain training, Ageing, Self-care, Enhancement, Neuroscience, Users

## Abstract

The use of ‘brain training’ games is often regarded as relating to wider ideals of self-improvement and youthfulness. Hence, use is intertwined with discourses of ‘active’ ageing. This paper analyzes how the use and users of brain training games were configured in the UK media, from 2005 to 2015, and examines how notions of active ageing relate to these representations. Game users were rarely constructed solely as gamers, and were more often presented as prudent individuals focused on a serious goal. This configuration related to assumed and enjoined motivations for brain training; specifically, users were commonly framed as seeking to enhance cognition and limit/delay cognitive decline. Scientific evidence about brain training was often deployed to explain how games might work; sometimes, however, it was used to undermine the utility of games and assert the significance and cognitive health-benefits of other activities. A minority of texts explicitly critiqued ideals of self-improvement, arguing that game playing was important for its own sake. Yet, even the pleasure associated with gaming was occasionally instrumentalized as a mechanism for ensuring prudent life choices. The analysis casts fresh light on how debates around health, ageing, and science correspond to configurations of technology uses and users. It presents evidence of the widespread cultural circulation of enjoiners regarding self-care and healthy ageing within British society. However, the paper also provides indications of the limits to such imperatives: discourses of pleasure co-exist with and perhaps supplant logics of prudence in (accounts of) practices ostensibly aimed at ageing ‘well’.

## Highlights

• This article analyses UK newspaper coverage of brain training games from 2005 to 2015.
• It interrogates how the uses and users of brain training games are configured.
• It examines these configurations against a backdrop of active ageing and self-care.
• Scientific evidence is drawn on in coverage to support and to refute brain training.
• Coverage links to responsibilization but challenges it through a discourse of pleasure.

## 1 Introduction

Within many societies, the meaning of ageing seems to be changing. Notably, ‘active ageing’ (i.e., “the process of optimizing opportunities for health, participation and security in order to enhance quality of life as people age” (WHO, 2002, 12)) has become a proxy for good ageing. Ageing well is not only something people do when they are old. Rather, individuals across much of the life-course are encouraged to eat healthily and stay fit: to undertake prudent practices of self-care that will maintain “oneself in a non-diseased state for as long as possible” (Pickard, 2011, 326; Lamb, 2014). This relates to a more general imperative to live, and stay, ‘well’ (Higgs et al., 2009). Hence, discourses concerning active ageing and health promotion intertwine and reinforce one another.

Computerized ‘brain training’ games are devices or software that purport to improve cognition. Promoted as one way to ameliorate the (cognitive) ageing process, games often focus on enhancing arithmetic and cognitive processing skills (e.g. memory) (Owen et al., 2010). Through game design and marketing, manufacturers draw on scientific ideas about neurological plasticity and cognitive development and deterioration - as well as wider notions of healthy ageing and fears about age-related decline (such as dementia). The use of computer games (including brain training) has extended to demographics previously less concerned with gaming technologies, such as older adults (Jones and Thiruvathukal, 2012, Juul, 2012; see also De Schutter et al., 2015). Some scholars have explicitly linked brain training to cultural narratives of active ageing, which they regard as placing undue pressure on older adults (Millington, 2012).

Enthusiasm for brain training has been noted in various countries, with popular and academic science articles reflecting on the associated ‘hype’ (e.g. George and Whitehouse, 2011, Yong, 2016). These have been critical of some of the claims made about its effectiveness, with various researchers studying or commenting on the evidence in this regard (e.g. a 2014 Consensus Statement). The scientific basis for brain training can today perhaps best be regarded as inconclusive (Owen et al., 2010, Papp et al., 2009), although one recent discussion of brain plasticity and training in *Nature Reviews Neuroscience* noted that the cumulative evidence for generalised or transferred effects on cognition resulting from one trained skill was weak (Lindenberger et al., 2017).

Scientific interventions into debates on brain training support on-going discussion in popular spheres. In doing so, they also enhance connections between news media, academic forums, and civil society. The BBC, the Alzheimer's Society, and the Medical Research Council (MRC), for instance, partnered in 2015 to test the effectiveness of brain training (Roberts, 2015). The focus was on the effects of games on memory and reasoning in older adults. Such endeavours underscore how demand and interest in brain training seem to reflect wider conversations, assumptions, and fears about cognitive decline and dementia (George and Whitehouse, 2011).

In the UK, the traditional news media has engaged extensively with brain training games (e.g. we located 336 items between 2005 and 2015 across six newspapers). In it, issues like enhancement, ageing, fun, and health are diversely conceptualised and discussed. Such reporting at once undergirds and propels societal engagement with brain training, and is the focus of our analysis. Specifically, we analyze how writers in UK newspapers imagine and articulate the users of computerized brain training games, and the uses to which games are put.

As Smirnova argues, “it is important to interrogate those various media outlets that perpetuate and reify various codes related to “the will to health”” (Smirnova, 2012: 1239). By analyzing accounts of brain training in the British press, we add empirical weight to claims made regarding how brain training supports cultural ideals of self-care and active ageing (Pitts-Taylor, 2010, Thornton, 2011). Accordingly, we cast fresh light on the ubiquity of those ideals per se by examining how our data support - and suggest limits to - assumptions about the dominance of discourse around self-care in British society. By interrogating brain training, we also contribute to debates around ageing (cf. Jones and Higgs, 2010, Higgs et al., 2009, Katz, 2000, Pickard, 2011, Williams et al., 2012), and the cultural circulation of neuroscientific knowledge (Broer and Pickersgill, 2015; Choudhury et al., 2010; Pickersgill, 2013, Pitts-Taylor, 2010).

### 1.1 Age, ageing, and the brain

Within discourses of active or healthy ageing, efforts to pursue health are actions to which moral worth adheres (Higgs et al., 2009, Millington, 2012). Such pursuit can be supported by technology, which is promoted as one way of postponing ageing and remaining independent. From drugs to walking aids to surveillance technologies, older adults increasingly “interact with science and technology to negotiate health and illness” (Joyce and Loe, 2010, 171–172). Consumer products (such as brain training devices) that pertain to arrest or even ‘cure’ aspects of ageing are frequently found within popular media, including through advertisements that can “both promise and normalize expectations of eternal youth” (Smirnova, 2012: 1236).

The concept of ‘functional age’ has become increasingly important as a measure complementing or even replacing chronological age (Katz and Marshall, 2004, Pickard, 2011). Moreira (2015) has demonstrated the key place of functional age within biomedicine, and has further noted a similar trend in popular discourses (e.g. in internet tests promising people the answer to the question: ‘what is your real age?’) (Moreira, 2016). This notion underscores conceptions of age and ageing as things people can (be expected to) more or less influence to maintain health and vigour (Jones and Higgs, 2010, Mykytyn, 2006). Techniques and tools for affecting cognitive vitality – promoted in the media and elsewhere – include the ‘training’ of brains (Lawless and Augoustinos, 2017; Thornton, 2011).

The brain, as well as its functions (i.e. cognition), is increasingly interpolated within discourses on ageing (Williams et al., 2011) as “something of a ‘project’ [of self-care] in its own right” (Williams et al., 2012, 67). In particular, policy discourse has come to emphasize the importance of taking care of one's brain in (and in advance of) later life, as a means of staving off age-related decline (and especially dementia) (Broer and Pickersgill, 2015). This emphasis relates to wider understandings of self-care wherein working on the brain is deemed central to maintaining a state of (optimized) normality throughout the life-course (Brenninkmeijer, 2010, O'Connor and Joffe, 2015, Rose, 2007).

Scholars like Katz and Peters (2008, 349) have viewed objects and ideals aimed at enhancing and maintaining cognitive health as reflecting a “hypercognitive society that expects infallible, anti-age-able cognitive skills”. Discourse around brain plasticity - i.e., the notion that the structure and function of the brain can change over time, either by accident or design - has been understood by some social scientists to extend expectations such as these and to support an “ethic of personal self-care and responsibility” (Pitts-Taylor, 2010, 639; see also Millington, 2012, Thornton, 2011). Indeed, brain health more generally has been shown to be constructed as an individual responsibility within the media (Lawless and Augoustinos, 2017). Here, we layer empirical complexity upon assertions made around brain training per se, and self-care more generally. In illustrating the shifting configurations of uses for and users of these technologies, we simultaneously evidence and - to an extent - challenge assumptions about ‘hypercognitive’ societies (e.g. Katz and Peters, 2008, Williams et al., 2012).

## 2 Methods

Our analysis is informed by science and technology studies (STS) insights regarding how (imagined) technologies, users, and practices are co-configured (see in particular Woolgar, 1990). We focus on the print news media, since this is “likely to play an important role in shaping public perceptions of new technologies and their value and applications” (Coveney et al., 2009: 488). Hence, configurations of users within the media have implications for how practices and values are constituted in practice. Nevertheless, we are mindful of the divergence between the neuroscientific ideas presented within texts and the perspectives expressed by individuals (O'Connor and Joffe, 2015, Pickersgill et al., 2015), as well as how ‘traditional’ and ‘new’ media might configure issues differently.

Lexus-Nexis was used to search for articles on computerized brain training games in six major UK newspapers. Our attention to the UK relates to our broader concerns with how neuroscientific knowledge is translated and leveraged within a variety of British policy and popular contexts, and is analyzed in the context of our wider work in this area (e.g., Broer and Pickersgill, 2015, Pickersgill, 2013, Pickersgill et al., 2011, Pickersgill et al., 2015). Both broadsheets (*Daily Telegraph*, *Times*, *Guardian*) and tabloids (*Daily Mail*, *Sun*, *Mirror*) were included. Search terms were, first, ‘brain training’, and, then, combinations of ‘game’, ‘improve’ and ‘brain’. Searches were restricted to items published from 1st January 2005 (the launch year of the seminal Nintendo DS game, ‘Dr. Kawashima's Brain Training’) until 31st December 2014. The ten-year period examined produced a manageable yet meaningful sample. Mentions of ‘brain training games’ were less frequent from 2011 onwards, with just 15 articles published in 2015, which thus represented the end-point for our analysis (see Table 1).

**Table 1:** A description of the source and year of the articles in our core data set.

	2005^a^	2006	2007	2008	2009	2010	2011	2012	2013	2014	**Total:**
The Sun	–	10	7	6	8	3	4	1	2	3	**44**
The Guardian	–	15	11	6	9	2	3	–	3	6	**55**
The Observer	–	4	4	4	2	2	2	1	1	1	**21**
Daily Telegraph	–	3	16	7	6	15	–	1	3	2	**53**
Daily Mail	–	4	7	11	7	5	2	1	2	2	**41**
Mail on Sunday	–	1	–	1	–	2	–	–	–	–	**4**
Times	–	9	13	18	10	6	2	2	2	1	**63**
Sunday Times	–	4	8	5	4	4	2	1	–	–	**28**
The Mirror	–	2	5	6	7	5	2	–	–	–	**27**
**Total:**	**-**	**52**	**71**	**64**	**53**	**44**	**17**	**7**	**13**	**15**	**336**

a The only article on brain training in 2005 is one that is part of our contextualising dataset, and does not mention computerized brain training games.

Media items were scanned by Broer for relevance, and we selected those that referred explicitly to brain training computer games (most commonly, those produced by Nintendo) marketed as a means of enhancing cognition (n = 336 articles). Even when brain training games were mentioned largely tangentially, the article was included in the analysis since we regarded casual mentions as important in playing a role in configuring game uses and users. Items were more often found in broadsheets than tabloids (220 versus 116 items). Table 1 details the source and publication date of items included in this data set. Although puzzles like Sudoku were often referred to as brain training, we did not analyze articles mentioning these in depth (n = 151). Nevertheless, they helped to contextualise our primary data and we inspected them to better comprehend the broader discourse of brain training.

Data were organised using Nvivo. A combination of deductive and inductive approaches were used within our constructivist analysis: we interrogated the data with particular ideas in mind, based on the literature (e.g. regarding the links between self-care, enhancement, and brain training), as well as taking cues from grounded theory approaches which are more orientated towards the location of unanticipated themes. Initial coding stayed close to the meaning of the specific fragments of text, which meant first distinguishing between sometimes similar-sounding codes such as ‘exercising one's brain’ and ‘mental workout’. This produced over 240 codes, with similar codes then grouped into 18 broad categories to make better analytic sense of the data (e.g. science and evidence; enhancement; fun, pleasure, and enjoyment; the origins of the effectiveness of brain training). We ultimately focused on three key issues containing much of the data mapped to the original 18 themes: the configuring of uses; the configuring of users; and, science and self-care. For instance, ‘the configuration of uses’ contains many of the metaphors and descriptions of brain training use, as well as notions like enhancement.

In what follows, we first examine the various kinds of uses assumed and anticipated for brain training games. Next, we analyze how users of brain training games are configured in the media as, especially, prudent and reflexive. The focus in the proceeding section turns to how evidence for brain training games was employed in journalists' accounts. The Discussion returns to the diverse discourses shaping the rhetorical machinery configuring technology users. We attend especially to ambivalences in the imaginaries of uses and users of brain training games, and the debates around health, ageing, and science that these propel. Throughout the article we largely refer to those writing in newspapers as ‘journalists’, although some might be better considered ‘commentators’. We do not regard journalists as a homogeneous group, and have borne authorship in mind during our analysis, as well as where in newspapers the items analyzed were situated.

### 2.1 Configuring uses

The backdrop to many articles on brain training games was often that these were a form of entertainment that could potentially have serious benefits. As an *Independent* interview with Ryuta Kawashima (i.e. Nintendo's ‘Dr Kawashima’) of Tohoku University noted, brain training “games are more than just a fun way to learn: they could, in fact, provide a revolutionary new way to treat dementia” (*Independent*, 27/05/13, Science). Brain training games were sometimes heralded for their effects on users' cognitive skills (e.g. working memory, capacity for multi-tasking, attention span, and intelligence), with the benefits of this enhanced functioning portrayed as extending to other areas in life. As one *Sunday Times* (25/06/06, Features) piece put it: “By training your brain, you're making a commitment to maintain your intelligence, and by doing that, you're likely to be open to new ideas, possibilities and inventions.” The use of brain training games, then, did not just develop cognition or arrest its decline – it enhanced subjectivity more broadly.

The term ‘training’ within the phrase ‘brain training’ offered up many opportunities for comment (cf. Millington, 2012, O'Connor and Joffe, 2015, Pitts-Taylor, 2010). Journalists regularly presented the brain as a kind of muscle, with brain training games said to ‘strengthen’ and ‘stretch’ brains:

The exercises, based on brain-scanning research, are designed to activate as many regions of your prefrontal cortex as possible, strengthening neural connections and even creating new ones. This is all just neuroscience-speak for the weird sensation - it actually feels almost like a literal stretching of the mind - you get when you attempt one of Kawashima's favourites, the Stroop test. (*Guardian*, 31/03/07, Guardian Weekend).

As in aerobic exercise, discomfort was framed as a sign of the effectiveness of brain training, with ease and enjoyment regarded as an indication to switch exercises and to increase intensity:

After four days of 20 min doing the dual n-back [a brain training game], I have no idea if it's working, but it's definitely hurting. Sadly, that's probably a good sign, and it's one thing on which researchers do tend to agree: if intelligence can be boosted by brain games - a very big if - they almost certainly won't be enjoyable ones. Unless the task involved keeps getting harder, so that you never quite feel you've got the hang of it, there's no way you'll get more intelligent. (*Guardian*, 04/01/14, Guardian Weekend).

Brain training games were thus linked with fitness regimens that emphasize the pain felt on the way to enhanced speed, strength, and vitality. Such rhetoric configured the use of brain training games as an extension of pre-existing practices of self-care and improvement, of the kind embedded within discourses of active ageing (cf. Katz, 2000).

So common was an implicit framing of the brain as muscle - even in the early years of brain training - that it was flagged in one article as a truism, and in part rejected: the brain is “not, as the brain trainers like to say, a muscle. It is a 1.3 kg crème caramel-like mix of fat, water and proteins driven by electricity and chemicals called neurotransmitters” (*Sunday Times*, 16/11/08, Features). Yet, a similar understanding of revivification was nevertheless put forward in the same article, with a scientist quoted to reinforce the point:

So your brain may be rotting, but there should, in theory, be something you can do to keep it reasonably fresh. The important concept here is “brain plasticity”, the ability of the brain to change and adapt. “We are literally remaking our brains,” writes [Nancy C.] Andreasen in The Creative Brain, “- who we are and how we think, with all our actions, reactions, perceptions, postures, and positions - every minute of the day and every day of the week and every month and year of our entire lives.” (*Sunday Times*, 16/11/08, Features).

Such quoting of scientists was not uncommon, as in the following from an article noting how neurological “deterioration can be held in check”:

'If you lead a full and active life, take new interests and learn new skills, you can increase the number of connections between your brain cells,' says psychologist Susan Blackmore. 'It really is a case of use it or lose it.' (*Daily Mail*, 04/11/08, Health).

Interviews with scientists were not necessarily used to explicitly advocate for brain training, and often served to advance wider commentary on the science of learning and cognition in ways that often – as above – emphasized the biosocial benefits of active ageing.

A few articles on brain training in schools and prisons could be read as more critical accounts of the use of games in these contexts. Since this was a marginal trope in the data, this analysis does not explicitly focus on it. Rather, games were most often presented (in broadsheets and tabloids alike) as being used for enhancing citizens' brains and their selves, and to arrest or stave cognitive decline (see Millington, 2012, Pitts-Taylor, 2010). By using games in this way, wider benefits were often deemed accruable. Framing the use of brain training as a means of augmenting cognition and limiting decline contributes to the configuration of a user who, though potentially enjoying gaming, is also prudently focused on exercising their brains to improve their health and well-being. Sensitive to the extent to which conceptions of uses and users dynamically relate to one another, in the next section we explore the configuration of users in more depth. We will also analyze some of the ambivalences and ambiguities conveyed through this discourse.

### 2.2 Configuring users

Many articles provided a narrative of the rise of brain training as occurring first in Japan, then in the UK. Nintendo's marketing strategy was noted in at least 24 (primarily broadsheet) items. Responding to promotional campaigns, some articulated similar stories about actress Nicole Kidman's role advertising the Nintendo DS system. The marketing move was said to be aimed at promoting “the console's appeal beyond its traditional young male audience” (*Daily Mirror*, 25/06/07, News).

Accounts of the popularity of brain training often underscored the ‘new’ users employing them, and of computer games more generally; for instance, by claiming 2007 “was the year computer games burst out of the geek's bedroom and into the living room” (*Sunday Times*, 16/12/07, Features). The Nintendo Wii console and its portable DS system gradually appeared as suggested family pastimes or presents, such as for “Mums” (e.g. the Wii Fit system; *Daily Mail*, 11/12/09, TV and Showbiz) and “Grandparents” (e.g. the Nintendo DS Lite, “including the popular Brain Training”; *Daily Telegraph*, 11/12/10, Features). While teenage boys were anticipated to enjoy more vivid games like ‘Mortal Kombat (*Times*, 17/12/07, Features), older relatives would apparently take (a more serious kind of) pleasure in gentler gaming experiences like brain training.

Older adults’ use of gaming technologies was explained in part by the words ‘brain training’ in software titles, and a desire from users to augment cognition or limit decline:

Nintendo DS games have been marketed as “brain training” – focusing on a serious purpose rather than play. Although research has shown that the Wii Fit isn't as effective as going to the gym, and there's little evidence that brain training can help slow mental decline, the “training” packaging helps to sell these games to a demographic that might be unwilling to buy something purely for fun. (*Guardian*, 13/05/11, Features).

Hence, journalists often assumed that the (older) users of brain training games were not necessarily ‘traditional’ gamers.

The games were regularly presented as part of a larger general self-improvement market, focused on making people appear and feel younger:

You've sorted your gym membership, your personal trainer and your Botox. But how can you hope to retain that sparkle of youth unless you've signed up for a decent brain-fitness regime? As mortality looms for the Baby Boom generation, businesses are piling in to market new mental-stimulation programmes that claim to slow the brain's rate of decline. From computer gaming to publishing, the new “brain training” promises to enhance mental agility and stave off dementia, using the latest neuroscience to stretch thinking power. At a time when we have ever-increasing expectations of our senior years, it's a compelling sales pitch. (*Times*, 06/05/06, Times Magazine Features).

Journalists were sometimes critical of a discourse of self-improvement, and of the marketing of games. As Victoria Coren wrote in an ironic commentary for *The Observer*:

One of the commercials explains that the brain-training machines are “inspired by the research of Dr Kawashima”. What a brilliantly opaque, advertising-style sentence. The elderly target's subliminal mind inhales “research” and “doctor”, so she comes away thinking this is a development akin to the discovery of penicillin. But if you actually think about it, the words “inspired by” mean almost nothing at all. I could perform a contemporary dance inspired by the research of Dr Kawashima and that wouldn't stave off Alzheimer's either. (*Observer*, 15/02/09, Observer 7Days).

Such irony and sarcasm was common within the articles analyzed. They were perhaps most apparent when authors discussed (and criticized) the ‘brain age’ calculated by the Nintendo ‘Dr Kawashima’ game. A popular trope was journalists' disclosure of ‘alarm’ regarding their own brain age, followed by a personal story of using the game, and reflections on the experience. For example, Oliver Burkeman (a *Guardian* journalist who has written various articles about brain training and the desire to be young) described how:

The first time I subjected myself to Dr Ryuta Kawashima's brain-ageing technology, I learned that I had the brain of a 51-year-old. I mean no disrespect to 51-year-olds when I say I found this alarming. I was planning on waiting a couple of decades before turning 51. All of us are in cognitive decline from early adulthood onwards. But how come I'd jumped so far along the road so quickly? I was jet-lagged, I protested. But Kawashima - who's a real person, but who appears in the Brain Training computer program as a disembodied bouncing head - just chuckled. He chuckles a lot, no doubt because he possesses the brain of a 20-year-old, which is what he says we should all be aiming for. (*Guardian*, 31/03/07, Guardian Weekend Pages).

The playfully articulated horror of discovering one has a brain that is apparently older than one's chronological age often rested on the notion that the brain declines from age 20. Burkeman, for instance, noted that we are all “in cognitive decline from early adulthood onwards”. It is the wide cultural circulation of conceptions of post-adolescent decline - and the fears associated with it - that, according to journalists, has contributed to the popularity of brain training, and to the expansion of the demographic group playing computer games. Such concerns are similar to those articulated within media accounts of brain health and training in contexts outside the UK (Lawless and Augoustinos, 2017, Thornton, 2011).

Users of brain training games were frequently configured as sensible, future-thinking, and reflexive, employing games for rational ends. While a discourse of self-care was apparent both in tabloid and broadsheet newspapers, it was particularly emphasized in the latter, with tabloid newspapers more likely to mention celebrities such as tennis player Andy Murray playing brain training games (e.g. *Sun,* 24/01/09, Sports). Across the news items analyzed, users were commonly imagined to be older than ‘traditional’ gamers; however, younger people too were framed as wanting to enhance their cognition or to stave off decline (or simply to relax). They were generally presented as having awareness of the negative aspects of ageing, comprehension of some of the neurocognitive research giving support to their concerns, a desire to respond to these worries earlier in the life-course, and the necessary capabilities - both resources and will - to act in ways that would ameliorate (the effects of) aging. In this context, the optimizing effects of brain training in earlier life buttressed the brain against decline at a later point.

By configuring users of brain training games as individuals who wish to age actively and healthily, the UK media connects game use and users to widely made enjoiners to care for the (ageing) self ever earlier in life (Williams et al., 2012). Simultaneously, however, the framing of prudent game use was at times related to a justification of the assumed pleasures found in it (as in the extract from the *Guardian* article at the beginning of this section) (cf. Thornham, 2009). In what follows, we analyze how scientific evidence was deployed within accounts of brain training, and cast further light on how articulations of pleasure complicate the dominant message of prudence.

### 2.3 Science and self-care

As indicated earlier, articles discussing brain training sometimes communicated the science associated with games. The names of brain regions, for instance, were occasionally included alongside an explanation of their functions (e.g. the pre-frontal context, “used for learning and thinking, expressing personality and moderating social behaviour - in other words […] the part of the brain that makes us human” (*Times*, 29/09/07, Features). An article in *The Times* employed a neurobiological idiom when explaining the (in)famous Dr Kawashima's perspectives on brain training as a means of preventing, or at least slowing, cognitive decline. As in some other media items, this framed the potential users of brain training not as specifically older adults, but adults in general:

[Dr Kawashima] claims to have devised a series of exercises that increase delivery of oxygen, blood and amino acids to the prefrontal cortex, the region of the brain that makes up much of the frontal lobe, which is responsible for creativity, memory, communication and self control. The programme is designed for those suffering from forgetfulness, difficulty remembering names, how to spell words or express thoughts, or who wish to work on creativity, memory skills, communication and slowing the mental effects of ageing. That would be all of us then. (*Times*, 15/02/07, Features).

However, both broadsheets and tabloids commented too on the lack of scientific support for game use as a means of extending cognitive skills or limiting decline. As one researcher quoted in *The Daily Mail* (27/01/09) stated: “The Nintendo DS is a technological jewel. But it is charlatanism to claim that it is a scientific test”. Ambivalence and cynicism were evident in 2006 and 2007, albeit less emphasized, but scepticism was particularly present in 2009 (with at least 15 references to a lack of evidence) - and in 2010 (12 references), when the BBC and a team of researchers tested the effectiveness of brain training. Ultimately, the games failed; as one *Daily Mirror* (21/04/10, Features) piece put it: “People who used the brain training games fared no better than others who surfed the internet for a similar amount of time on sites such as Wikipedia.” Dementia specialist Clive Ballard, of King's College London and the Alzheimer's Society, was quoted in the *Daily Mirror* and the *Daily Mail*, for instance, as stating that “staying active by taking a walk, for example, is a better use of our time”. (*Daily Mail*, 21/04/10, Science News). Quotes such as these underline, as well as dash, hopes that brain training can act as a means of enhancing cognition in the present in order to arrest or limit its decline in the future.

A similar position to Ballard's was argued for even more forcefully in the following discussion about a brain training game called the ‘dual n-back’:

You might feel inclined to stick to brain games instead, on the rationale that even if they don't work, they can't do any harm. But that position's arguably misguided. Your time is finite, and every hour you spend wrestling with the dual n-back is one you could have spent doing any of the more mundane things that will certainly promote brain health: doing sufficient physical exercise, getting enough sleep, and preparing and eating healthy food. “Live a good clean life, get proper sleep and you'll be at the peak of whatever your potential performance is,” James Thompson suggests. (*Guardian*, 04/01/14, Guardian Weekend).

Hence, rejection of brain training games could itself be considered a responsible form of self-care, provided time was spent on other beneficial activities. Time is presented here as a resource that should be used prudently, resonating with the kinds of responsibilities enjoined in wider calls to live and age actively and healthily (Lamb, 2014, Lawless and Augoustinos, 2017, Thornton, 2011, Williams et al., 2012).

Notably, another - albeit less visible - discourse focused more on the pleasure that brain training games could bring. At least four (broadsheet) articles explicitly addressed this in relation to scientific evidence, although engagement with the topic of fun and enjoyment in general was more widespread in the media items examined. We discuss it here as a negative case; i.e., a pattern in the data that diverges from the overall pattern. For instance, in the following quote commentator Victoria Coren asserted that publicising research demonstrating no cognitive benefits from brain training elided the pleasures of gaming:

This all feels to me like pouring cold water, unnecessarily, on a harmless new fad that gave hope and purpose to people who felt their mental skills might be fading. Rushing to publicise the narrow, negative findings is mean-spirited and cynical. It's not like they were advocating a better way, they were just telling people not to do something fun. “Thought you were staving off the Alzheimer's with your silly little machine, did you, Grandpa? Ha! Think again, you hoodwinked old fool.” (*Observer*, 25/04/10, Comment).

Oliver Burkeman similarly wrote that “Since it feels so true, “brain exercise” makes people feel happier”. He concluded: “the best ways to care for your brain are probably physical exercise, good diet and sleep; but happier people are surely more likely to make those choices. (*Guardian,* 07/08/10, Guardian Weekend pages). Such prose can be read as an alternative means of perceiving the effectiveness of brain training games, as well as a different way of looking at (evidence-based) self-improvement. Pleasure in this context propelled self-care, but was also important per se.

In communicating science through writings on brain training, journalists further configured audiences in relation to wider discourses of neuroscience, plasticity, and optimization (Brenninkmeijer, 2010, Broer and Pickersgill, 2015, O'Connor and Joffe, 2015, Pickersgill, 2013, Pickersgill et al., 2015, Pitts-Taylor, 2010, Rose, 2007), citizens' responsibilities to self-care (Higgs et al., 2009, Katz and Peters, 2008, Millington, 2012, Williams et al., 2012, Thornton, 2011), and fears of cognitive decline (Lawless and Augoustinos, 2017, Peel, 2014). Some articles urged readers to use brain training games because if they failed to employ (indeed, optimize) their cognitive skills they would lose them. During the same time period, others contrarily asserted that the lack of evidence for brain training meant that people could or should make better use of their time. These articles reinforced the prudence of users of brain training by presenting them as the kinds of subjects who might ultimately reject games to find more effective means of self-care. Finally, a small number of items foregrounded the import of play, regardless of the scientific credibility of brain training. By emphasising the mundane pleasures of brain training, they destabilised the dominant configuration of users as prudent individuals instantiating an ethic of self-care within everyday life. Nevertheless, pleasure was itself on occasion instrumentalized as a mechanism through which to enjoin users to engage in practices associated with healthy, active, and happy ageing.

## 3 Discussion

Through analysing UK media accounts of brain training games, this article interrogated how inter-related discourses of self-care, ageing, and science operate within widely read texts. Hence, it adds further empirical depth to sociological examinations of these themes. As media studies scholar Roger Silverstone (2005: 200) has noted, media technologies (which for us includes newspapers) convey “the values, rules and rhetorics of their centrality for the conduct of the quotidian”. In effect, journalists configure themselves as authoritative sources regarding how readers are and should be living their lives. Accordingly, although we recognise the disjunctures that can exist between media texts and individual perspectives (O'Connor and Joffe, 2015, Pickersgill et al., 2015), we also maintain that newspaper accounts of the uses and users of technologies matter. These rhetorical productions have implications for readers' engagements in their everyday lives, since real-life use comes to be situated against (and so shaped by) the context of the imagined employment of brain training in the wider population. Hence, newspaper accounts of technology use continue to have relevance for the politics and practices of health.

We found that imagined and enjoined uses and users of brain training were constituted through a discursive matrix comprising shifting facets of ageing, self-improvement, gaming, and science (with journalists reflecting - sometimes critically - on these different concepts). A configuration of prudent use, orientated towards self-care, was commonly (but not exclusively) apparent throughout the years 2005–2015, and in both broadsheets and tabloids. This was somewhat more emphasized in the first five years, with later years increasingly focused on the (lack of) efficacy of brain training. Any pleasures assumed to be elicited through game use were largely articulated within more dominant tropes in which users were constructed as concerned primarily with the worthy pursuit of cognitive health and enhancement. This finding supports studies of gaming technologies that have shown that adult game use might not be regarded as age-appropriate (De Schutter et al., 2015), and hence rationalised and legitimizing in ways that reflect cultural norms of adulthood (Thornham, 2009).

With the use of brain training presented as relating to a desire to maintain or even enhance cognition, game users were largely presented as individuals who valued their cognitive skills and had the desire and will to better them – or to arrest or limit their decline. Such people might not normally buy games, but were sufficiently focused on self-care and improvement to engage with brain training despite the association of gaming with entertainment. Similar representations of self-care and improvement were apparent within the media accounts of cognitive health, decline, and optimization analyzed by other social scientists, including outside the UK (Lawless and Augoustinos, 2017, O'Connor and Joffe, 2015, Peel, 2014, Thornton, 2011). The cognitive benefits deemed accruable from game use also meant that some journalists presented (potential) users as any adult, with age-related decline framed as addressable prior to users becoming ‘old’ (i.e., cognitive enhancement earlier in life was constructed as staving off decline at a later point) (Williams et al., 2012). This finding resonates with Pitts-Taylor's study of popular coverage of neuroplasticity, where, a “moral pressure” to self-care also applied to “healthy subjects who have no known illness or complaint” (Pitts-Taylor, 2010: 646).

Practices of self-improvement were propelled through metaphorical language presenting the brain as a kind of muscle (see, relatedly, Millington, 2012, Pitts-Taylor, 2010). Linking brain training to wider discourses of fitness contributed to a conception of ageing as something amenable to intervention across the life-course (rather than a bodily process passively experienced). In so doing, media reporting on brain training potentially contributes to a wider reworking of the temporalities and ontologies of ageing (Armstrong, 1995, Pickard, 2011). Specifically, deleterious ageing was both brought forward (in particular, starting from age 20 onwards) and rendered something that could be postponed. Hence, reporting on brain training appears to underwrite a notion that “later life is something that can and should be prepared for earlier in life, not just because of the negative effect on the individual of not doing so, but because it is also now culturally appropriate to do so” (Williams et al., 2012: 66).

A discourse of neurobiological structure and cognitive function was apparent in explanations of the mechanisms and worth of brain training games. This relied upon and contributed to a wider cultural understanding of the brain as playing a role of (mundane) significance for people's sense of self (Pickersgill et al., 2011, Rose, 2007). Connecting scientific narratives to stories of ageing (and intimations of the malcontents associated with this) further positioned game use within a regime of prudent self-care, health-maintenance, and optimization. This contributed to the configuration of the individual training their brain more as a responsible user rather than a leisurely gamer, though these framings were not mutually exclusive.

The media items examined also provided illustrative cases of journalists deploying irony or sarcasm regarding self-improvement generally and/or brain training specifically. Such commentaries again highlight the cultural availability and significance of scripts around self-improvement into which brain training can be plotted, as well as the extent that these can be subject to resistance. One of the main points of critique for broadsheet commentators related to the joylessness of self-improvement for its own sake. For instance, some writers argued that there was nothing wrong with having fun through brain training, and more generally the presumed enjoyment of playing games on occasion served as a backdrop to the articles we examined. However, fun was itself sometimes seen as self-care too. By instrumentalizing pleasure as a means of legitimizing it, the dominance of enjoiners to age actively and healthily is underscored even as they were seemingly resisted (Higgs et al., 2009; Katz 200; Katz and Peters, 2008, Lamb, 2014, Williams et al., 2012).

Millington (2012: 429) has argued that brain training can “exacerbate the pressure on older persons to demonstrate an obvious ‘will to health’ through on-going consumerism” (and relatedly Pitts-Taylor, 2010; Thornton, 2011). Our findings suggest the need for hesitancy before fully embracing such concerns (see also O'Connor and Joffe, 2015). As our study shows, media accounts of brain training act to communicate imperatives to self-care, yet discourses pertaining or connecting to everyday pleasures imply limits to these. Digital cultures scholar Helen Thornham has argued that among UK adults, “there is still a perceived necessity not only *to* justify gaming, but to justify it as something *other* than pleasure, escapism or entertainment” (Thornham, 2009: 142). Hence, it is not unfeasible that culturally-sanctioned discourses of prudence circulate precisely to legitimize the practices of pleasure associated with brain training games, and hence play a role in the domestication (Silverstone and Hirsch, 1992; De Schutter et al., 2015) of these technologies by non-traditional gaming populations.

## 4 Conclusion

Accounts of brain training commonly emphasize brain plasticity and the related possibilities of cognitive enhancement (Millington, 2012, Pitts-Taylor, 2010) and the limiting of decline (Lawless and Augoustinos, 2017). They connect as well as with wider accounts of brain health and active aging, which often underline individual responsibility to self-care (Broer and Pickersgill, 2015, Katz and Peters, 2008, Peel, 2014, Thornton, 2011; Williams, Katz and Higgs, 2011). We have found that imperatives to care for one's ageing brain are evident within commentary and reporting on brain training games within UK media. Game use is often configured as prudent, with users presented as individuals (sensibly) concerned with ageing actively through attention to their brains. However, games also serve as a pivot from which journalists sometimes critique these concerns and practices; for instance, through a sarcastic inflection, the deployment of scientific evidence about cognition, and/or an argument for the import of the everyday pleasures of game use. Accordingly, while our data lend some support to assumptions and arguments about the cultural traction of discourses around active aging and self-care, it also points to the potential limits to these: pleasure seems to co-exist with and can perhaps supplant the logics of health and enhancement associated with practices of active ageing. Despite a well-documented “moral pressure” to attend to the self (Pitts-Taylor, 2010: 646), there is good reason for sociological suspicion that having fun is at least as important as taking care.

## Author note

Broer and Pickersgill contributed equally to this article, and hence should be considered joint first authors.
